# Supplementary material for: Injury-induced cold sensitization in Drosophila larvae involves behavioral shifts that require the TRP channel Brv1
Source: PLoS One. 2018 Dec 26;13(12):e0209577. doi: 10.1371/journal.pone.0209577 (PMC6306221; doi:10.1371/journal.pone.0209577)
Supplement: S1 Fig — (A) Proportion of US (red), CT (green), or BR (grey) responses to cold probe at different time points after UV, data shown as a stacked graph. For each behavior, the average over three sets of 30 larvae are shown. (B,C) Proportion of CT, US or BR fast (dark grey), slow (light grey) or non-responders (nr, white) in mock or UV-treated larvae to the cold probe (10°C) (B) 16 hours (C) or 24 hours after UV. Fast = response less than 4 seconds, slow = response between 4–10 seconds, nr = no response within 10 seconds. (D) Proportion of US, CT, or BR responses to the cold probe at different temperatures in mock or UV-treated larvae, data shown as a stacked graph. For each behavior, the average over three sets of 30 larvae are shown. (A-D) n = 3 sets of 30. Data are presented as mean ± s.e.m percentage of categorical responders. Stats: Two-tailed Fisher’s Exact test, * = p < 0.05, * = p < 0.001, comparisons were made between UV and mock control at each time point. (PDF) [file pone.0209577.s001.pdf]

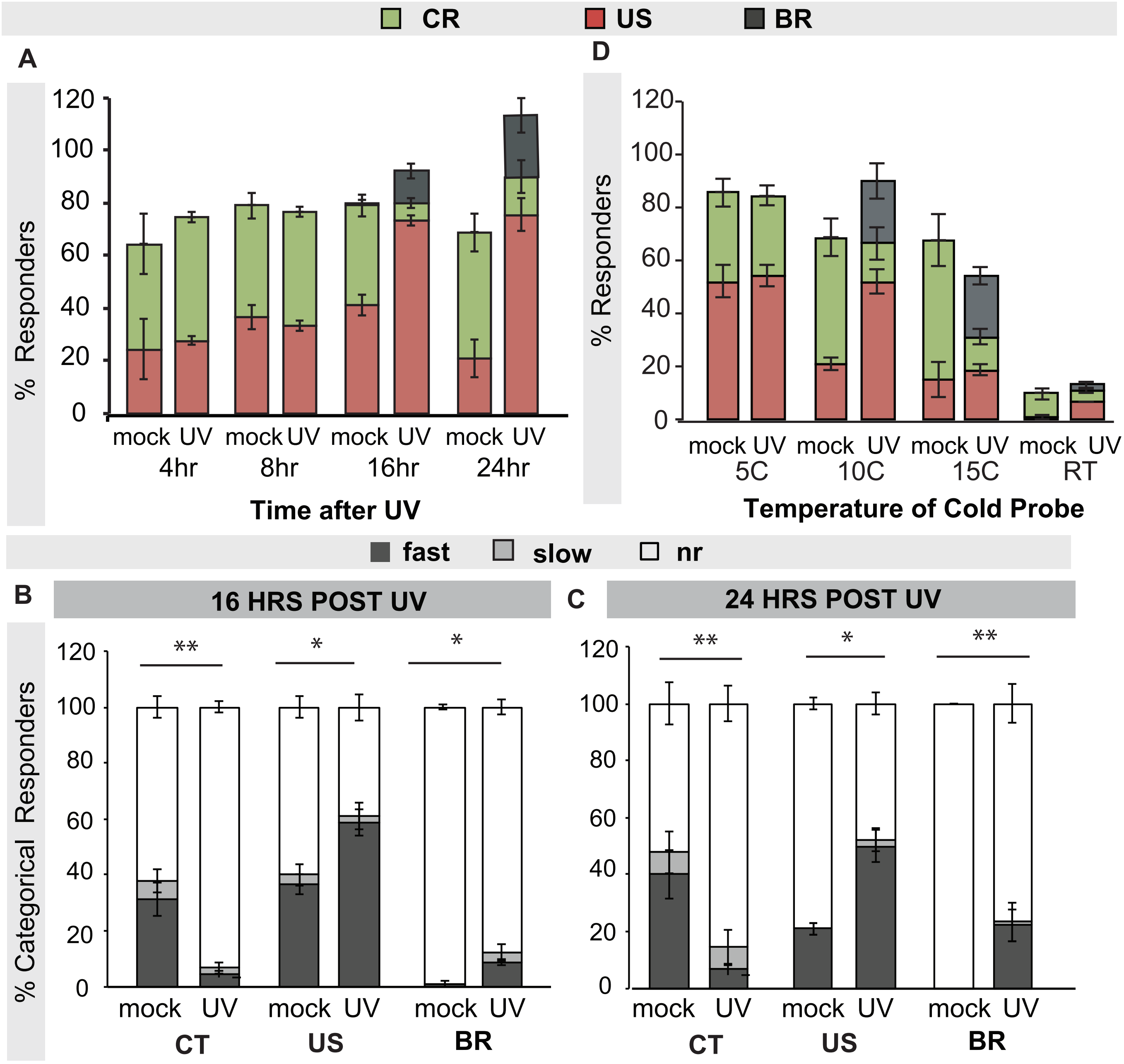

S1 Fig. Characterized responses to cold probe in mock- or UV-treated larvae. (A) Proportion of US (red), CT (green), or BR (grey) responses to cold probe at different time points after UV, data shown as a stacked graph. For each behavior, the average over three sets of 30 larvae are shown. (B,C) Proportion of CT, US or BR fast (dark grey), slow (light grey) or non-responders (nr, white) in mock or UV-treated larvae to the cold probe (10 ºC) (B) 16 hours (C) or 24 hours after UV. Fast = response less than 4 seconds, slow = response between 4-10 seconds, nr = no response within 10 seconds. (D) Proportion of US, CT, or BR responses to the cold probe at different temperatures in mock or UV-treated larvae, data shown as a stacked graph. For each behavior, the average over three sets of 30 larvae are shown. (A-D) n = 3 sets of 30. Data are presented as mean ± s.e.m percentage of categorical responders. Stats: Two-tailed Fisher's Exact test, * = p < 0.05, * = p < 0.001, comparisons were made between UV and mock control at each time point.
