# Supplementary material for: Injury-induced cold sensitization in Drosophila larvae involves behavioral shifts that require the TRP channel Brv1
Source: PLoS One. 2018 Dec 26;13(12):e0209577. doi: 10.1371/journal.pone.0209577 (PMC6306221; doi:10.1371/journal.pone.0209577)
Supplement: S2 Fig — Percent of responders to cold probe (10°C) 24 hours after UV with varying dose (10–14 mJ/cm2). Bars represent average responders ± s.e.m.. * = p < 0.05 by two-tailed Fisher’s Exact test, comparing percent responders of each behavior between each UV dose, both US and BR were significantly different at 13 mJ/cm2 when compared to other UV-doses n = 3 sets of 30. (PDF) [file pone.0209577.s002.pdf]

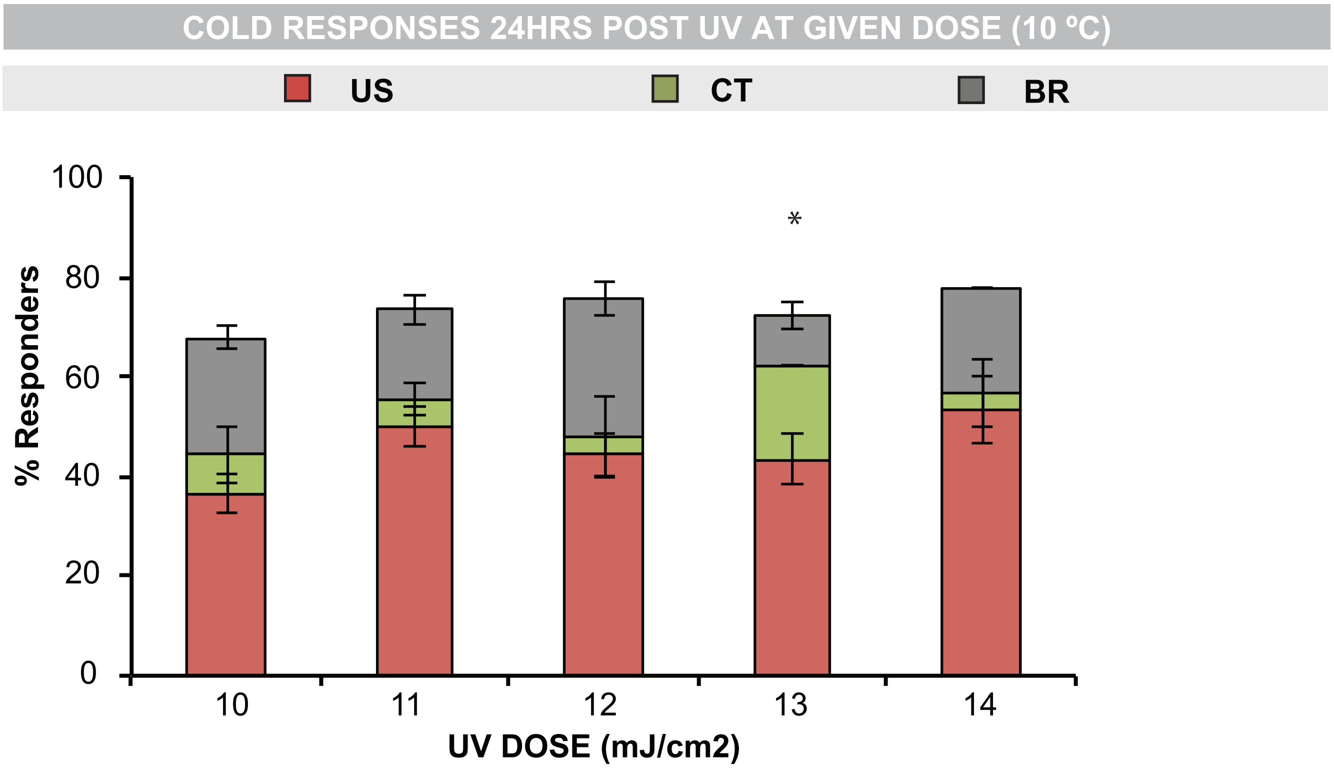

S2 Fig. Varying UV-dose has little effect on cold sensitization. Percent of responders to cold probe (10 ºC) 24 hours after UV with varying dose (10-14 mJ/cm2). Bars represent average responders ± s.e.m.. * = $p < 0.05$ by two-tailed Fisher's Exact test, comparing percent responders of each behavior between each UV dose, both US and BR were significantly different at 13 mJ/cm2 when compared to other UV-doses n = 3 sets of 30.
