# Supplementary material for: Injury-induced cold sensitization in Drosophila larvae involves behavioral shifts that require the TRP channel Brv1
Source: PLoS One. 2018 Dec 26;13(12):e0209577. doi: 10.1371/journal.pone.0209577 (PMC6306221; doi:10.1371/journal.pone.0209577)
Supplement: S3 Fig — (A-C) Percent change in GCaMP6m fluorescence at 10°C for mock- and UV-treated larvae 24 hours post-irradiation for CIII (A), Ch (B), and CIV sensory neurons (C), where the middle line is mean ± s.e.m. and n = 8–11 larvae. Stats: Two-tailed t-test (A-C), where the comparisons are between mock and UV treated conditions. n.s. = not significant. (PDF) [file pone.0209577.s003.pdf]

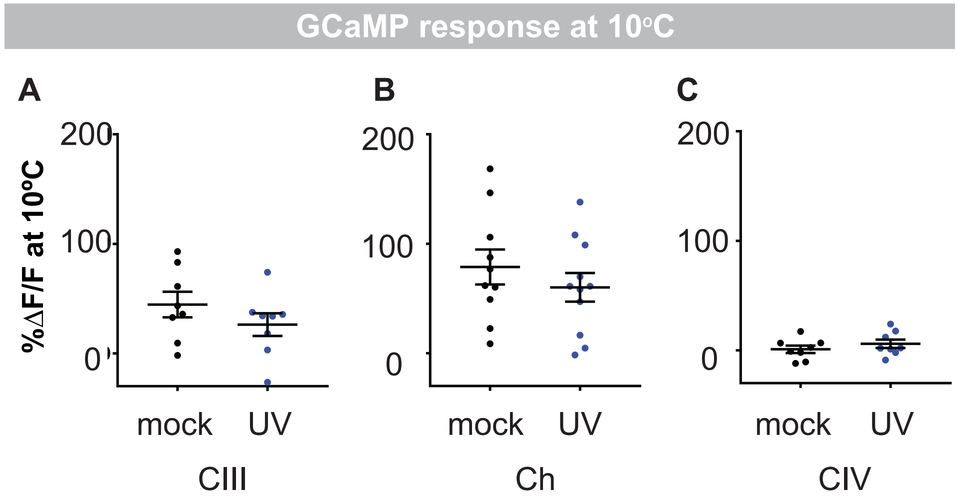


S3 Fig. UV irradiation does not alter cold-evoked calcium responses at 10°C. (A-C) Percent change in GCaMP6m fluorescence at 10°C for mock- and UV-treated larvae 24 hours post-irradiation for CIII (A), Ch (B), and CIV sensory neurons (C), where the middle line is mean ± s.e.m. and n= 8-11 larvae. Stats: Two-tailed t-test (A-C), where the comparisons are between mock and UV treated conditions. n.s. = not significant.
