# Supplementary material for: Injury-induced cold sensitization in Drosophila larvae involves behavioral shifts that require the TRP channel Brv1
Source: PLoS One. 2018 Dec 26;13(12):e0209577. doi: 10.1371/journal.pone.0209577 (PMC6306221; doi:10.1371/journal.pone.0209577)
Supplement: S4 Fig — (A) CT or (B) US, responses in wildtype (w1118) or Brv1 mutant larvae, shown as a percent change in response after UV. (C) CT or (D) US responses in larvae expressing Brv-RNAi in class IV (CIV) or Chordotonal (Ch) neurons, compared to genetic controls (w1118 and Gal4 alone). (A-C) Data is calculated as (% UV responders—% mock responders)/ % mock responders. Therefore all bars that are above 1 indicate that the UV response was less than the mock response, and all bars below 1 indicate the UV response was more than the mock response, as indicated by arrows. (PDF) [file pone.0209577.s004.pdf]

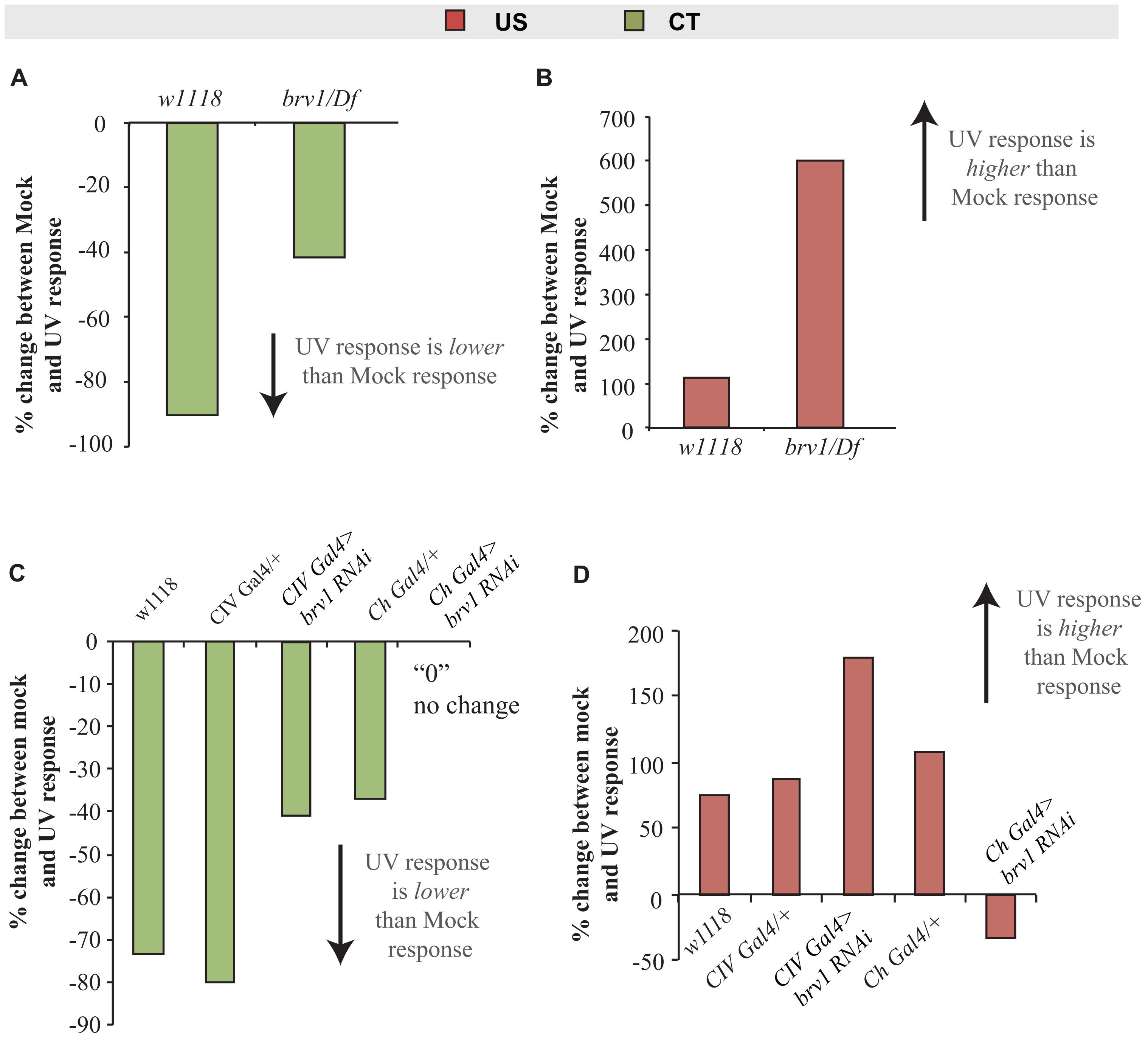

S4 Fig. Brv1 mutant and RNAi shown as percent change in response. (A) CT or (B) US, responses in wildtype (w1118) or Brv1 mutant larvae, shown as a percent change in response after UV. (C) CT or (D) US responses in larvae expressing Brv-RNAi in class IV (CIV) or Chordotonal (Ch) neurons, compared to genetic controls (w1118 and Gal4 alone). (A-C) Data is calculated as (% UV responders - % mock responders)/ % mock responders. Therefore all bars that are above 1 indicate that the UV response was less than the mock response, and all bars below 1 indicate the UV response was more than the mock response, as indicated by arrows.
